# Clinical efficacy and tolerability of zonisamide monotherapy in dogs with newly diagnosed idiopathic epilepsy: Prospective open‐label uncontrolled multicenter trial

**DOI:** 10.1111/jvim.17108

**Published:** 2024-05-23

**Authors:** Miyoko Saito, Akinori Nomura, Daisuke Hasegawa, Naoyuki Watanabe, Keiko Uchida, Seiichi Okuno, Masahiro Nakai, Kensuke Orito

**Affiliations:** ^1^ Laboratory of Small Animal Surgery (Neurology), School of Veterinary Medicine Azabu University Sagamihara Japan; ^2^ Department of Product Development Bussan Animal Health Co, Ltd Osaka Japan; ^3^ Laboratory of Veterinary Clinical Neurology, Graduate School of Veterinary Medicine Nippon Veterinary and Life Science University Tokyo Japan; ^4^ Watanabe Animal Hospital Shizuoka Japan; ^5^ AC‐plaza Kariya Animal Hospital Tokyo Japan; ^6^ Animal Clinic Kobayashi Saitama Japan; ^7^ Laboratory of Physiology II, School of Veterinary Medicine Azabu University Sagamihara Japan

**Keywords:** antiepileptic drug, antiseizure medication, canine, clinical trial, seizure, treatment

## Abstract

**Background:**

Zonisamide (ZNS) is a newer generation antiseizure medication (ASM) used to treat epilepsy in dogs and cats. However, scientific and clinical information, particularly regarding monotherapy, is limited.

**Objectives:**

To evaluate the antiseizure efficacy and tolerability of ZNS monotherapy in dogs with newly diagnosed idiopathic epilepsy (IE).

**Animals:**

Study included 56 client‐owned dogs newly diagnosed with IE.

**Methods:**

This was a prospective multicenter, open‐label, uncontrolled study. All dogs were ASM‐naïve and had ≥2 seizures within 12 weeks. Dogs were administered 2.7‐14.4 mg/kg ZNS PO q12h and followed up for ≥12 weeks. Data from the 12‐week maintenance treatment period were compared with those from the 4‐ to 12‐week pretreatment period for efficacy evaluation. Data from the entire ZNS administration period were used to assess tolerability.

**Results:**

Fifty‐six dogs were included in our study. Of the dogs, 53 were assessed for efficacy; 40 (76%) had a ≥ 50% reduction in seizure frequency, and 29 (55%) achieved seizure freedom. For 90% of the dogs with ≥50% reduction in seizure frequency, the mean ZNS dose was 4.8 (range, 2.7‐8.6) mg/kg q12h and the mean trough plasma ZNS concentration was 18.9 (range, 8.0‐48.0) μg/mL. In 7 of the 56 dogs (13%), reduced activity, decreased appetite, vomiting, hindlimb weakness, soft stools, or constipation was observed, albeit mild and temporary. Laboratory tests revealed no relevant changes.

**Conclusions and Clinical Importance:**

Our study suggests that ZNS monotherapy is effective and well‐tolerated in dogs with newly diagnosed IE.

**Abbreviations**
ASM: antiseizure medication
EEG: electroencephalography
IE: idiopathic epilepsy
IVETF: International Veterinary Epilepsy Task Force
KBr: potassium bromide
LEV: levetiracetam
MRI: magnetic resonance imaging
PB: phenobarbital
WBC: white blood cell
ZNS: zonisamide

## 1 INTRODUCTION

Zonisamide (ZNS; 1,2‐benzisoxazole‐3‐methanesulfonamide) is a newer‐generation antiseizure medication (ASM) used to treat epilepsy in dogs and cats. Zonisamide was 1st synthesized in 1979^1^ and has been available for human use as an ASM since 1989 in Japan and the early 2000s in the United States and Europe. Zonisamide has a broad spectrum of activity with multiple sites of action and is thought to exert its antiseizure effects primarily by inhibiting sustained, repetitive neuronal firing through blockade of voltage‐gated sodium channels and voltage‐gated T‐type calcium channels,^2^, ^3^ and as such is commonly categorized as an “excitation‐inhibiting ASM.” Other actions include enhancing GABA_A_ receptor function,^4^ inhibiting glutamate release,^5^ and protecting neurons by scavenging free radicals.^6^

Zonisamide demonstrates favorable pharmacokinetic properties in dogs and cats, with steady‐state concentrations achieved in as little as 5 to 7 days, and blood concentrations maintained with 12‐hour dosing.^7^, ^8^, ^9^, ^10^ Preclinical studies performed in dogs during the development of the drug for human use demonstrated its efficacy in suppressing experimentally induced seizures^11^, ^12^ and its long‐term safety.^13^ In Japanese veterinary medicine, ZNS's potential efficacy was 1st described by Ueda at an academic conference in 1996 and has been used as a relatively common ASM in dogs and cats based on its pharmacokinetic properties, lack of cytochrome p450 enzyme induction and favorable therapeutic index (see Supporting Information 1 for more details).^7^, ^8^, ^10^, ^13^, ^14^ In 2014, ZNS (Consave) was licensed for treating idiopathic epilepsy (IE) in dogs, making it the 1st ASM approved for veterinary use in Japan (see Supporting Information 2 for more details). Currently, ZNS is not approved for use in dogs or cats in either the United States or Europe and is typically employed as a 2nd‐ or 3rd‐line option.

To date, information on the clinical efficacy of ZNS as a treatment for epilepsy is limited in veterinary medicine. Two open‐label studies have explored the efficacy of ZNS as an add‐on treatment in dogs with drug‐resistant IE.^15^, ^16^ Moreover, ZNS monotherapy was evaluated in 1 study involving 10 dogs with newly diagnosed IE.^17^ As a result, the American College of Internal Medicine Small Animal Consensus Statement on Seizure Management gave ZNS a low recommendation as monotherapy and a moderate recommendation as add‐on treatment, based on the limited published research on epileptic dogs.^18^ Subsequently, caregiver‐assessed evaluations of clinical outcomes for ASM monotherapy, including ZNS, were published.^19^ Commonly reported adverse events of ZNS in dogs include ataxia, sedation, vomiting, and decreased appetite, which all tend to be mild and transient.^15^, ^16^, ^17^, ^19^ Acute or chronic hepatopathy,^20^, ^21^, ^22^ erythema multiforme,^23^ keratoconjunctivitis sicca,^15^ renal tubular acidosis,^24^, ^25^ urinary calculi,^22^ and aggressive behavior^26^ are considered rare. Possible association with ZNS administration has been reported in the arthropathy of 1 Lyme disease‐infected dog.^15^

We hypothesized that ZNS is a suitable ASM for monotherapy in canine IE. Our study aimed to evaluate the antiseizure efficacy and tolerability of ZNS in a multicenter, open‐label, uncontrolled study of dogs with newly diagnosed IE. Furthermore, we sought to determine the appropriate dosage and therapeutic range of ZNS when used as monotherapy. Our study formed part of the basis for ZNS, marketed under the trade name Consave, to be approved by the Japanese government for the treatment of IE in dogs.

## 2 MATERIALS AND METHODS

### 2.1 Study design and ethics

This was a prospective, multicenter, open‐label, uncontrolled study to evaluate the efficacy and tolerability of ZNS as monotherapy in dogs with IE. Our study was designed according to the Guidelines for Good Clinical Practice (GCP) for the Conduct of Clinical Trials of Veterinary Medical Products in Japan.^27^ Participation was voluntary, and informed consent was obtained from all owners before enrollment. Our study complied with the GCP approved by the Japanese Ministry of Agriculture, Forestry and Fisheries.^27^

### 2.2 Case selection

According to the National Veterinary Assay Laboratory notification,^28^ the number of animals necessary for the study was determined to be approximately 60. This predetermined sample size was not based on a power calculation. Client‐owned dogs with IE were recruited at 18 institutions (3 referral centers and 15 primary care practices) in Japan between 2009 and 2012. Eligible dogs were enrolled in the order in their presentation if they fulfilled the inclusion criteria. Specifically, dogs that were ASM‐naïve and had experienced at least 2 epileptic seizures during the 12‐week period before the study were considered eligible. IE was diagnosed based on a minimum Tier I confidence level, as described by the International Veterinary Epilepsy Task Force (IVETF).^29^ In addition, if the seizure onset was at <1 year of age, preprandial and postprandial bile acid testing was mandatory. Magnetic resonance imaging (MRI) and cerebrospinal fluid analyses were mandatory in dogs with seizure onset <1 year or >6 years of age, and only those with normal results were included. Electroencephalography (EEG) was performed in dogs with focal seizures that did not secondarily generalize, and only dogs with apparent epileptiform discharges in the EEG were included in our study.

### 2.3 Study protocol

#### 2.3.1 Efficacy

Our study consisted of a 4‐ to 12‐week baseline period, a dose‐titration period, and 12‐week evaluation period. Although the baseline period is generally set at 12 weeks, it was reduced to a minimum of 4 weeks for cases with a seizure frequency of more than once every 2 weeks. After the baseline period, ZNS tablets (25‐ or 100‐mg Consave tablets, Bussan Animal Health Co, Ltd, Osaka, Japan) were administered PO at an initial dose of 2.5 to 6.25 mg/kg q12h with a minimum of one‐quarter tablet (see Supporting Information 3 for more details). This dosage was determined based on both pharmacokinetic data^30^ and an efficacy study conducted in dogs with experimentally induced seizures.^11^ During the titration period, if the seizure frequency, severity, or both had not improved compared with the baseline period, the dose was incrementally increased by approximately 2.5 to 5 mg/kg or to the appropriate dose calculated using the measured plasma ZNS concentration at 1‐ to 2‐week intervals until seizures resolved, up to a maximum of 15 mg/kg q12h while ensuring the plasma ZNS concentrations were not above the upper limit of the target range (approximately 40 μg/mL)^13^, ^30^ and no major adverse events were observed. If plasma ZNS concentrations (trough value) exceed 50 μg/mL or if signs of adverse effects potentially related to the dose, such as decreased appetite, gastrointestinal signs, or decreased activity, were observed and deemed necessary for dose reduction or withdrawal by the attending clinician, the dose was gradually tapered by a minimum of one‐quarter tablet until the signs disappeared to avoid withdrawal seizures. The rate of tapering was left to the discretion of each clinician depending on the individual's adverse effect status. The adjusted appropriate dose was maintained for 12 weeks during the evaluation period.

Plasma ZNS concentrations obtained immediately before dosing (i.e., trough) were measured by high‐performance liquid chromatography approximately 7 days after ZNS initiation, at each dose escalation, and at the end of the evaluation period. Historical seizure frequency, severity, and duration, and age of onset of the 1st seizure were recorded in medical records. Each owner was provided with a seizure log and asked to report the number, date, time, and duration of each seizure, the body part(s) involved in the seizures (at onset and as seizure progressed), and details of the dog's condition during and after the seizures. The seizure log was reviewed every 2 weeks by a clinician, and a new log was provided to the owners. Seizure types were classified according to the IVETF classification as focal epileptic seizures, generalized epileptic seizures, and focal epileptic seizures evolving into generalized epileptic seizures,^29^ based on the owner's descriptions and seizure video clips.

The primary efficacy endpoints of our study were changes in seizure frequency (seizures per month) and seizure‐free rate. Cluster seizures were defined as 2 or more seizures occurring within a 24‐hour period, with each seizure counted separately. Status epilepticus was defined as continuous seizures for more than 5 minutes or more than 1 discrete seizure without complete recovery of consciousness and was counted as a single seizure. The change in seizure frequency was assessed based on the number of seizures per month and the seizure frequency reduction rate. Four weeks was defined as 1 month. The number of seizures per month during the 12‐week evaluation period (E) was compared with that during the 4‐ to 12‐week baseline period (B). Subsequently, the seizure frequency reduction rate was calculated as 100 × (B−E)/B, where positive values indicate a reduction and negative values indicate an increase in seizure frequency compared with baseline. Dogs with seizure frequency reduction rates of ≥50% were considered to have responded to the treatment. Seizure‐free rate was the percentage of dogs who became seizure free. The other endpoint was the comparison of seizure severity between the baseline and evaluation periods. Severity was assessed based on the mean duration of a single seizure episode, number of cluster seizures, and status epilepticus.

#### 2.3.2 Tolerability

Tolerability of ZNS was evaluated in all dogs from the time of enrollment to study completion. Owners were asked to maintain a diary for daily recordings of medication administration time, appetite, level of activity, vomiting, abnormal stools, any adverse events or changes. This information was collected every 2 weeks with the seizure log and reviewed by the clinician. Physical and neurologic examinations were performed at each hospital visit. CBC, serum biochemical analysis (chemistry), and urinalysis were also evaluated upon enrollment, at the end of the study, and when a change in physical condition was reported. Additional tests were conducted at the clinician's discretion, if necessary. Regarding the frequency of hospital visit, dogs were seen every 1 to 2 weeks, but if seizures continue or there are changes in their condition, owners could bring them in for more frequent evaluations. As an exception, it is permitted to have a visit every 4 weeks, for the evaluation period only, if the diary and seizure log were evaluated by the clinician every 2 weeks.

### 2.4 Statistical analyses

Statistical analyses were performed using the commercially available software program MINITAB version 16 (Minitab Inc, State College, Pennsylvania, USA). Data were preliminarily analyzed for normality using the Anderson‐Darling test, and all data were found to be non‐normally distributed. In addition, given the small sample size, a nonparametric method was employed.

The Wilcoxon signed‐rank test was used to compare the median number of seizures per month, seizure duration, number of episodes of cluster seizures, and status epilepticus observed during the evaluation period with those during the baseline period, and body weights between enrollment and study completion. The Kruskal‐Wallis test was used to compare the median seizure frequency reduction rate among the seizure types. Because of its high sensitivity in detecting differences, a paired *t* test was utilized to compare laboratory results between enrollment and study completion to ensure safety. Statistical significance was established at *P* < 0.05 for all analyses.

## 3 RESULTS

### 3.1 Dogs

Fifty‐six dogs were enrolled in our study, including 30 males (10 castrated and 20 intact) and 26 females (16 spayed and 10 intact), with a median body weight of 5.6 kg (range, 1.6‐41.3 kg). The median age at enrollment was 53.5 months (range, 6‐156 months). The breeds represented were mixed breed (n = 6), miniature dachshund (n = 7), Yorkshire terrier (n = 6), toy poodle (n = 6), Chihuahua (n = 6), Pomeranian (n = 5), golden retriever (n = 2), papillon (n = 2), Shetland sheepdog (n = 2), French bulldog (n = 2), Siberian husky (n = 2), and 1 each of Irish setter, Italian greyhound, Pembroke Welsh corgi, Cavalier King Charles spaniel, great Pyrenees, Shiba Inu, schipperke, pug, beagle, and border collie.

Eight of the 56 dogs (14%) had focal seizures, 7 (13%) had focal seizures that secondarily generalized, 35 (63%) had generalized seizures, and 6 (11%) had both focal and generalized seizures, as determined from the detailed owner descriptions and video clips. Video during seizures was analyzed in approximately 50% of the dogs. Thirty‐two (57%) of the dogs had at least 1 episode of cluster seizures, 1 (2%) experienced 3 episodes of status epilepticus, and 1 (2%) experienced 1 episode of cluster seizures and status epilepticus.

### 3.2 Efficacy

Fifty of the 56 dogs completed the efficacy evaluation. Three were removed from the study during the titration period; 2 owners withdrew consent for reasons unrelated to medical matters, and the owner of the 3rd dog withdrew consent because of a perceived lack of therapeutic benefit during the titration period without further increasing the dose. No adverse events were reported in the 3 dogs. The remaining 3 dogs dropped out during the evaluation period. One dog was withdrawn on day 48 of the evaluation period after the owner requested a dose reduction. Although the dog's seizures were controlled, the owner reported that it had its 1st estrus cycle and developed behavioral changes characteristic of estrus. The remaining 2 dogs were withdrawn on days 15 and 23 of the evaluation period because of lack of efficacy. Therefore, efficacy data were obtained from a total of 53 dogs, consisting of the 50 dogs that completed the study and 3 dogs that were withdrawn from the study owing to lack of efficacy. The total ZNS administration period ranged from 13 to 40 weeks.

**FIGURE 1:**
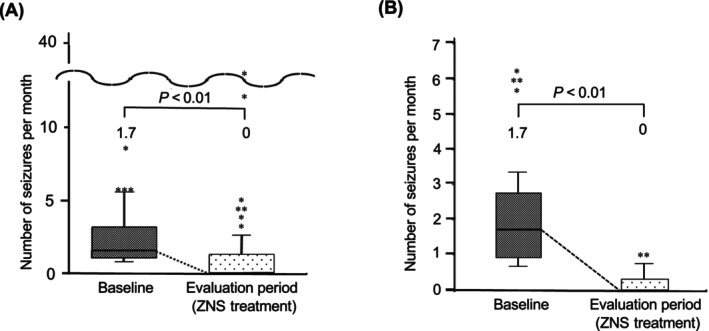
Comparison of seizure frequency (number of seizures per month) at baseline and evaluation period for dogs with idiopathic epilepsy administered zonisamide (ZNS) monotherapy in (A) all dogs and (B) responders. The bold horizontal line within the boxplot represents the median, the ends of the box represent the interquartile range, and the upper and lower whiskers extend 1.5 × interquartile range from the top and bottom of the box. The Wilcoxon signed‐rank test was used to compare baseline and evaluation periods.

The overall seizure frequency (seizures per month) decreased significantly in the evaluation period (median, 0.0) compared with baseline (median, 1.7; *P* < .01; Figure 1A), with a 100% (median) seizure frequency reduction. Seizure frequency decreased in 42 dogs and increased or remained the same in 11 dogs during the evaluation period compared with baseline.

Of the 53 dogs, 40 (76%) had a ≥50% reduction in seizure frequency compared with the baseline and were considered responders. Notably, 29 (55%) dogs achieved complete freedom from seizures during the 12‐week evaluation period. The seizure frequency (seizures per month) for all responders was decreased significantly during the evaluation period (median, 0.0) compared with baseline (median, 1.7; *P* < .01; Figure 1B), with a 100% (median) seizure frequency reduction. To determine the therapeutic dose and targeted plasma concentration, mean ZNS dose and trough ZNS concentration were calculated for 50%, 90%, and 100% of responders. For half of the responders, ZNS doses ranged between 2.7 and 4.9 mg/kg q12h. For 90% of responders, the mean ZNS dose was 4.8 mg/kg (range, 2.7‐8.6 mg/kg) q12h, and the mean trough plasma ZNS concentration was 18.9 μg/mL (range, 8.0‐48.0 μg/mL). For all responders, the mean ZNS dose was 5.5 mg/kg (range, 2.7‐14.4 mg/kg) q12h, and the mean trough plasma ZNS concentration was 21.9 μg/mL (range, 8.0‐64.8 μg/mL).

The remaining 13 dogs (24.5%), including the 3 that were withdrawn because of lack of efficacy, were nonresponders, showing a 25% increase in seizure frequency (a median seizure frequency reduction, −25%). For those dogs, the mean ZNS dose was 7.8 mg/kg (range, 5.1‐12.2 mg/kg) q12h, and the mean trough plasma ZNS concentration was 40.8 μg/mL (range, 12.7‐73.5 μg/mL). In 2 of the nonresponders, although no serious adverse events were observed and their plasma ZNS concentrations did not reach the upper limit, the ZNS dose was not increased up to 15 mg/kg q12h, because the duration of their seizures decreased and the owners were satisfied with the current ZNS treatment.

**FIGURE 2:**
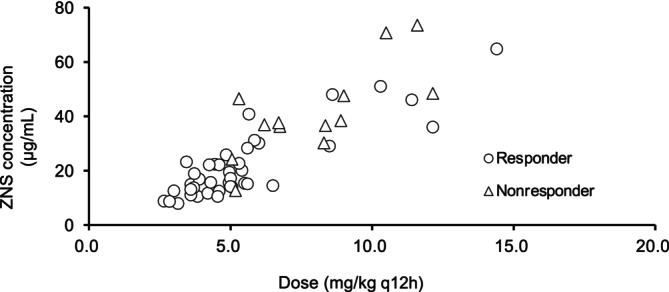
Zonisamide (ZNS) dose and trough plasma concentrations at evaluation period in 53 dogs. Large interindividual differences in trough plasma ZNS concentrations were observed.

Large inter‐individual differences in trough plasma ZNS concentrations for dogs during the evaluation period were identified (Figure 2). For example, at a ZNS dose of 4.6‐5.4 mg/kg q12h, plasma ZNS concentrations ranged from 10.6‐46.5 μg/mL.

Thirty‐five dogs experienced at least 1 episode of cluster seizures or status epilepticus during the baseline and evaluation period. The total number of cluster seizures and status epilepticus episodes decreased significantly during the evaluation period compared with baseline, with a median monthly frequency of 0.0 (range, 0‐2) and 0.3 (range, 0‐2), respectively (*P* = 0.005). In 25 of these 35 dogs (71%), the number of episodes of cluster seizures and status epilepticus in the evaluation period decreased by ≥50% compared with that of the baseline period. Among the 29 dogs that achieved seizure freedom, 2 had status epilepticus during the baseline period.

Six of the 13 dogs (47%) in the nonresponder group had a ≥50% reduction in the duration of a single seizure episode or number of episodes of cluster seizures and status epilepticus during the evaluation period compared with baseline.

There was no significant difference in median seizure frequency reduction among the 4 seizure types (*P* = .39): 100% for generalized epileptic seizures, 77% for focal epileptic seizures, 100% for both generalized and focal epileptic seizures, and 95% for focal epileptic seizures secondarily generalizing. The responder rate was 80%, 70%, 80%, and 57% in generalized epileptic seizures, focal epileptic seizures, both generalized and focal epileptic seizures, and focal epileptic seizures that secondarily generalized, respectively. The number of dogs that achieved seizure freedom was 21 out of 34 dogs with generalized epileptic seizures, 2 of 7 dogs with focal epileptic seizures, 3 of 5 dogs with both generalized and focal epileptic seizures, and 3 of 7 dogs with focal epileptic seizures that secondarily generalized.

### 3.3 Tolerability

Tolerability of ZNS was evaluated in 56 dogs that received at least 1 dose. Laboratory tests identified a significant increase in chloride (mean ± SD, 114.3 ± 4.1‐117.9 ± 5.7 mmol/L, *P <* 0.001), total cholesterol (199.8 ± 59.9‐217.2 ± 64.2 mg/dL, *P =* 0.002) and serum creatinine (0.94 ± 0.3‐1.03 ± 0.3 mg/dL, *P <* 0.001) and a significant decrease in potassium (4.4 ± 0.5‐4.2 ± 0.5 mmol/L, *P* = 0.0111) and alanine transaminase (49.5 ± 23.2‐41.3 ± 23.8 IU/L, *P* = 0.0088) at the end of the study compared with values obtained at the time of enrollment.

Chloride was above the reference range at study completion in 5 dogs (median, 125 mmol/L, range, 124‐127 mmol/L), whereas no such increases were seen at enrollment in these dogs (median, 115 mmol/L, range, 109‐120 mmol/L). Specifically, this increase in chloride concentration ranged from 1 to 15% (median, 2%) in the 5 dogs. No clinical signs were noted, and blood gas analyses performed in 2 of these dogs revealed no acidemia. Two others (chloride increases of 2% and 3%) showed mild gastrointestinal signs during the evaluation period, which resolved within 1 or 2 days such that it was considered unlikely that ZNS was the cause of the signs. No other clinical signs potentially related to hyperchloremia or acidemia were observed. Discontinuation or dosage reduction of ZNS was not required in any of the dogs.

Cholesterol concentrations were above the reference range at study completion in 1 dog (321 mg/dL, reference range, 107‐314 mg/dL), whereas there was no such increase at the time of enrollment (300 mg/dL). This dog developed mild diarrhea during the evaluation period that spontaneously resolved within 1 day without any treatment. Zonisamide was considered to unlikely be the cause. No other clinical signs of hypercholesterolemia were detected, and discontinuation or dose reduction of ZNS was not required.

Although the mean creatinine concentrations increased at study completion compared with that at the time of enrollment, all individual creatinine values were within the reference range throughout the study.

Potassium concentrations were below the reference range at study completion in 5 dogs (median, 3.6 mmol/L, range 3.3‐3.7 mmol/L), whereas no such abnormalities were observed at the time of enrollment (median, 4.1 mmol/L, range, 3.9‐4.5 mmol/L). None of these dogs had concurrent hyperchloremia. The degree of potassium decrease ranged from 5 to 27% (median, 15%). Three of these dogs had mild gastrointestinal signs (i.e., vomiting, diarrhea, and decreased appetite) during the evaluation period, and recovered completely within 1 day. No other clinical signs potentially related to hypokalemia were observed, and discontinuation or dosage reduction of ZNS was not required in any of the dogs.

Other abnormalities observed in the CBC or chemistry of individual dogs included transient leukopenia and hypertriglyceridemia in 1 dog each. Specifically, the white blood cell (WBC) count was 8900/μL (reference range, 6000‐17 000/μL) at the time of enrollment and 5280/μL at study completion in 1 dog. The WBC differential percentages were within the reference ranges, and the dog did not show any clinical signs of leukopenia and spontaneously returned to normal without intervention. Although the cause of this change was unclear, ZNS treatment was considered less likely. The dog was administered the same dosage of ZNS, and its WBC count was within the reference range for 5 years at the time of last follow‐up. In the other dog, serum triglyceride was 200 mg/dL (reference range, 17‐102 mg/dL) at the time of enrollment and further increased to 661 mg/dL at study completion. The dog was obese (body condition score, 4/5), and its weight did not change during the study period. It was concluded that hypertriglyceridemia was unlikely related to ZNS administration and weight loss, diet change, and retesting were advised. However, no weight loss was achieved, and the owner did not wish to retest the triglycerides.

In 49 dogs, urinalysis data were available from the time of enrollment and study completion. No statistically significant changes in parameters were observed. In the microscopic analysis of the sediment, struvite crystalluria was not present at the time of enrollment but was encountered during ZNS treatment in 3 dogs, whereas such crystalluria was present at the time of enrollment but was not encountered during ZNS treatment in another 2 dogs. No clinical signs of crystalluria or other relevant abnormalities were observed on urinalysis. No treatment was determined to be necessary, and ZNS was administered at the same dosage.

**FIGURE 3:**
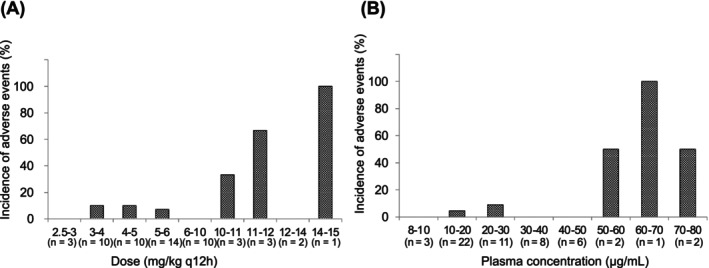
Relationship between the incidence of adverse events and dose (A) or trough plasma concentration (B) of zonisamide (ZNS). The figures depict ZNS doses and trough plasma concentrations when adverse events were observed. The incidence of adverse events was remarkably increased at doses ≥10 mg/kg q12h and trough plasma concentrations ≥50 μg/mL.

At least 1 adverse event was observed in 7 of the 56 dogs (13%) during ZNS treatment, which included reduced activity (n = 4), decreased appetite (n = 4), vomiting (n = 2), hindlimb weakness (n = 1), soft stools (n = 1), and constipation (n = 1). Because all adverse events were transient and mild, none of the dogs required discontinuation of ZNS treatment because of these events. Adverse events in 2 (4%) of these dogs were considered to be likely related to ZNS treatment. One dog had mild anorexia 2 days after starting ZNS, which resolved within 2 days without any intervention. The dog also showed mildly decreased activity after increase in ZNS. The dosage was returned to the preincrease dosage in 1 day at the discretion of the attending veterinarian 2 weeks after the dosage increase, resolving the clinical signs within 9 days. The other dog showed mild hind limb weakness 2 days after ZNS initiation, which disappeared spontaneously the following day. The remaining 5 dogs reportedly had undesirable events in which the causal relationship with the ZNS treatment was unclear, yet could not be denied. These were also considered adverse events, including decreased activity (n = 3), anorexia (n = 3), vomiting (n = 2), soft stools (n = 1), and constipation (n = 1). All these signs were mild and transient and either resolved spontaneously or after dose adjustment during the titration period. The evaluation of the relationship between dose or plasma concentration and adverse events revealed a notably high incidence of adverse events at doses ≥10 mg/kg q12h and at trough plasma concentrations ≥50 μg/mL (Figure 3).

Throughout the study period, none of the owners claimed that their dogs had an increased appetite. The body weight of 1 dog was not documented at study completion; therefore, weight change was evaluated with data from 55 dogs. Median body weight was not significantly changed (*P* = 0.60) at the study end (median, 5.7 kg, range, 1.7‐42.2 kg), compared with that at the study enrollment (median, 5.6 kg, range, 1.6‐41.3 kg).

## 4 DISCUSSION

Our study aimed to prospectively assess the effectiveness and tolerability of ZNS as a monotherapy for dogs with newly diagnosed IE. The findings suggest that the drug was efficacious and well‐tolerated in our study sample, with 40 dogs (76%) experienced a ≥50% reduction in seizure frequency compared with pretreatment, and 29 dogs (55%) achieved seizure freedom. The overall median reduction in the number of seizures (seizures/month) compared with pretreatment was 100%, with a mean of 52%. In addition, ZNS reduced seizure duration and showed effectiveness against all seizure types, including cluster seizures and status epilepticus, although larger studies might further clarify the efficacy by seizure type.

The study also aimed to determine an appropriate dose and therapeutic range for ZNS monotherapy. Because the majority of responder doses ranged from 2.7 to 8.6 mg/kg q12h and trough plasma concentrations from 8 to 48 μg/mL, and adverse events increased with doses above 10 mg/kg q12h or plasma concentrations above 50 μg/mL, we propose that the starting dose for ZNS as monotherapy for dogs with IE is 2.5‐5 mg/kg PO q12h, whereas the trough ZNS concentrations should be maintained at 10‐40 μg/mL. Remarkably, this therapeutic range aligns with the established range for humans.^31^

Our study demonstrated better potential efficacy compared with the previous study on ZNS monotherapy.^10^ The prior study was a single noncomparative, open‐label study involving 10 dogs with newly diagnosed IE. Among them, 6 (60%) were considered responders, with a ≥50% reduction in monthly seizure frequency compared with pretreatment. These responders were then monitored for a period of 12 to 36 months, during which 4 dogs (40%) achieved seizure freedom. In contrast, our findings revealed responder and seizure freedom rates of approximately 76% and 55%, respectively. This discrepancy might be because of differences in drug dosage. In our study, ZNS was increased stepwise during the titration period based on clinical signs and trough ZNS plasma concentrations, whereas the previous study did not measure trough values for all dogs, and the available trough values for nonresponders indicated the potential for further increasing the ZNS dose. Therefore, it is possible that the efficacy could have been further improved if the dose had been increased stepwise as in our approach. Other possible reasons include differences in the duration of the baseline and observation periods, differences in the drugs used, that is, dosage forms and excipients of drugs from different manufacturers, and differences in the study sample.

In the present study, common adverse events attributable to ZNS included reduced activity (n = 4), decreased appetite (n = 4), and vomiting (n = 2). Other notable adverse events included constipation (n = 1) and soft stools (n = 1). Because all the cases were transient and mild, none of the dogs discontinued ZNS treatment because of the adverse events. The adverse events of polydipsia and polyphagia, which are common in many other “inhibition‐enhancing” ASMs such as phenobarbital (PB) and potassium bromide (KBr), were not seen in the present study using ZNS, an “excitation‐inhibiting” ASM, as monotherapy. Other rare adverse events not seen in the present study but been reported include idiosyncratic reactions, such as hepatopathy, erythema multiforme, and keratoconjunctivitis sicca, as well as nephro‐urologic and behavioral changes.^15^, ^22^, ^23^, ^24^, ^25^, ^26^ Recognizing these rare events is important as these conditions can be fully reversible if the drug is quickly discontinued.

A correlation between plasma ZNS concentrations, particularly the maximum plasma concentration, and decreased appetite and reduced activity has been observed. Experimental studies conducted on dogs have demonstrated that the negative effects of decreased appetite can be mitigated by administering ZNS in 2 divided doses instead of a single daily dose, thereby reducing the maximum serum concentration.^13^ In our study, decreased appetite and activity were both fully reversible with dosage reduction.

Mild hyperchloremia was observed in 5 of 56 dogs after ZNS initiation. Reportedly, ZNS has weak carbonic anhydrase inhibitory activity, and the hypochloremia may be a compensatory effect of metabolic acidosis caused by carbonic anhydrase inhibition of ZNS in the renal tubules. However, because the potency of ZNS in this respect is approximately 1/100th of that of acetazolamide,^32^ whether this activity itself causes clinical problems is unclear. In our study, no acidemia was observed in dogs that had blood gases measured owing to increased chloride concentrations. Although ZNS treatment is considered a rare cause of metabolic acidosis, it should be used with caution in animals at risk for metabolic acidosis, such as those with impaired respiratory or renal function.

As a general rule, when seizures are not well controlled, a single ASM is preferred to be increased to the point where no adverse events occur, but if control is still not achieved, add‐on therapy is necessary. The concomitant use of ASMs that inhibit the excitatory system and those that enhance the inhibitory system have few overlapping adverse events. Therefore, this combination treatment is called rational multidrug combination therapy and is recommended as it is expected to enhance the antiseizure effects of a drug without increasing its adverse events. Because the main mechanism of action of many ASM available in veterinary medicine, such as PB, KBr, benzodiazepines, and imepitoin, is to enhance the inhibitory system, the presence of ZNS, which primarily inhibits the excitatory system, is important for rational multidrug therapy.

Our study had some limitations. A large‐scale randomized controlled trial (RCT) is best suited for drug evaluation, which was not performed here. Since the efficacy and tolerability of ZNS monotherapy, which may be sufficient as a 1st‐line ASM, has been demonstrated in the uncontrolled study presented here, it would be of particular interest to next conduct a RCT comparing ZNS with other 1st‐line ASMs. The baseline period for our study was short. This could falsely increase the baseline seizure frequency. The evaluation period was also relatively short: 12 weeks and did not follow outcome; therefore, the efficacy may differ in the longer‐term. The IVETF recommends a minimum 24‐week evaluation period in clinical studies.^33^ The IVETF also recommends assessing behavioral comorbidities and quality of life of dogs and caregivers.^33^ Because our study was initiated before that IVETF recommendation was published, we did not specifically evaluate these parameters, but owners were instructed to note any changes. A recently published study compared the clinical outcomes of dogs treated with levetiracetam, ZNS, and PB monotherapybased on caregivers' survey responses.^19^ This study reported that all 3 ASMs showed improved quality of life, with ZNS demonstrating the lowest occurrence of adverse events.

In conclusion, our findings demonstrate that ZNS monotherapy may be effective and well‐tolerated in dogs with IE. Hence, ZNS appears to be a suitable 1st‐line ASM for canine IE, and its addition among the 1st‐line ASMs is anticipated to improve epilepsy treatment in veterinary medicine. Further randomized controlled studies are needed to more accurately assess the safety and efficacy of ZNS monotherapy in dogs with IE.

## CONFLICT OF INTEREST DECLARATION

Drs Saito, Hasegawa, Watanabe, Uchida, Okuno, and Orito have received funding from Bussan Animal Health Co, Ltd within the last 10 years for some or all of the following activities: research, travel, speaking fees, consultancy fees, and preparation of educational materials. No authors received compensation for their publication activities [Correction added on 4 June 2024 after first online publication: The sentence has been amended.]. The company has no right to make publishing decisions. Drs Nomura and Nakai were formerly employed by Bussan Animal Health Co, Ltd.

## OFF‐LABEL ANTIMICROBIAL DECLARATION

Authors declare no off‐label use of antimicrobials.

## INSTITUTIONAL ANIMAL CARE AND USE COMMITTEE (IACUC) OR OTHER APPROVAL DECLARATION

Approved at each investigator site by the Ethics Committee. Written informed consent was obtained from the owners for the participation of their animals in this study. The study conduct was approved by the national authority in Japan (Regulatory Rules for Veterinary Medicinal Products, December 24, 2004, Ordinance of the Ministry of Agriculture, Forestry and Fisheries No. 107).

## HUMAN ETHICS APPROVAL DECLARATION

Authors declare human ethics approval was not needed for this study.

## Supporting information


**Data S1.** Supporting Information.
**1.** A few more details about the pharmacokinetics, therapeutic index, and pharmacodynamic characteristics of zonisamide.
**2.** The status of zonisamide approval for dogs in Japan.
**3.** The details of prospective initial dose determination for each dog in the study protocol.
