# Supplementary material for: Clinical efficacy and tolerability of zonisamide monotherapy in dogs with newly diagnosed idiopathic epilepsy: Prospective open‐label uncontrolled multicenter trial
Source: J Vet Intern Med. 2024 May 23;38(4):2228–36. doi: 10.1111/jvim.17108 (PMC11256125; doi:10.1111/jvim.17108)
Supplement: Supplementary file 1 — Data S1. Supporting Information. 1. A few more details about the pharmacokinetics, therapeutic index, and pharmacodynamic characteristics of zonisamide. 2. The status of zonisamide approval for dogs in Japan. 3. The details of prospective initial dose determination for each dog in the study protocol. [file JVIM-38-2228-s001.pdf]

SUPPORTING INFORMATION

1. A few more details about the pharmacokinetics, therapeutic index, and pharmacodynamic characteristics of zonisamide (ZNS).

The time to peak concentration, peak concentration, and elimination half-life after a single oral dose of 5-20 mg/kg in dogs are reported to be approximately 3-7 hours, 14-20 μg/mL, and 13-17 hours, respectively.[1-3] In cats receiving a single oral dose of 10 mg/kg, these values are similarly reported to be approximately 4 hours, 13 μg/mL, and 30 hours, respectively.[4] In terms of the therapeutic index, the ED50 for dogs from our present study is 5 mg/kg/dose, and the LD50 for dogs from previous studies is reported to be 1000 mg/kg/dose.[5] Therefore, the therapeutic index of ZNS in dogs is estimated to be 200. Regarding the pharmacodynamic characteristics of ZNS, unlike most first-line ASMs in veterinary medicine that primarily exert their effects by enhancing inhibition, ZNS primarily suppresses excitation through blockade of voltage-gated sodium channels and voltage-gated T-type calcium channels.[6,7]

2. The status of ZNS (Consave®) approval for dogs in Japan.[8]

ZNS (Consave®) is licensed for treating focal seizures (including secondary generalization) and generalized seizures in canine idiopathic epilepsy.

Dosage and administration

The usual starting dose of zonisamide is 2.5–5 mg/kg orally approximately 12 hours apart. Thereafter, titrate as necessary based on clinical signs. Following dose escalation, the usual dose is up to 10 mg/kg per dose.

Dosage forms

Consave® Tablet: 25 mg, 100 mg

3. The details of prospective initial dose determination for each dog in the study protocol.

Appropriately sized tablets or portions of tablets were not specifically used. Instead, to ensure a single dose within the range of 2.5 to 6.25 mg/kg, owners were dispensed either 25 mg or 100 mg tablets, or tablets divided into halves or quarters.

References

1. Matsumoto K, Miyazaki H, Fujii T, et al. Absorption, distribution and excretion of 3-(surfamoyl [^{14}C]methyl)-1,2-benzisoxazole (AD -180) in rats, dogs and monkeys and of AD-810 in men. Arzneim.-Forsch./Drug res. 1983;33:961-968.
2. Orito K, Saito M, Fukunaga K, et al. Pharmacokinetics of zonisamide and drug interaction with phenobarbital in dogs. J Vet Pharmacol Ther. 2008;31:259-264. doi:10.1111/j.1365-2885.2008.00955.x
3. Boothe DM, Perkins J. Disposition and safety of zonisamide after intravenous and oral single dose and oral multiple dosing in normal hound dogs. J Vet Pharmacol Ther. 2008;31:544-553. doi:10.1111/j.1365-2885.2008.00993.x
4. Hasegawa D, Kobayashi M, Kuwabara, et al. Pharmacokinetics and toxicity of zonisamide in cats. J Feline Med Surg. 2008;10:418-421. doi:10.1016/j.jfms.2008.01.006
5. Takemoto Y, Senda H, Yamazoe H, et al. Toxicity study of new antiepileptic drug Zonisamide (AD-810) - (I) Acute toxicity study in mouse, rat, dog, monkey and juvenile rat -. Jpn Pharmacol Ther. 1987;15(11):4337-4346. Japanese
6. Schauf CL. Zonisamide enhances slow sodium inactivation in *Myxicola*. Brain Res., 1987; 413:185-188. doi:10.1016/0006-8993(87)90168-5
7. Suzuki S, Kawakami K, Nishimura S, et al. Zonisamide blocks T-type calcium channel in rat cultured cerebral neurons. Epilepsy Res., 1992;12:21-27. doi:10.1016/0920-1211(92)90087-a
8. The package insert for Consave®, revised in June 2023 (4th edition)
